# Supplementary material for: Developmental mRNA m5C landscape and regulatory innovations of massive m5C modification of maternal mRNAs in animals
Source: Nat Commun. 2022 May 5;13:2484. doi: 10.1038/s41467-022-30210-0 (PMC9072368; doi:10.1038/s41467-022-30210-0)
Supplement: Supplementary file 1 — Supplementary Information [file 41467_2022_30210_MOESM1_ESM.pdf]

Supplementary Information for

Developmental mRNA m^5C landscape and regulatory innovations of massive m^5C modification of maternal mRNAs in animals

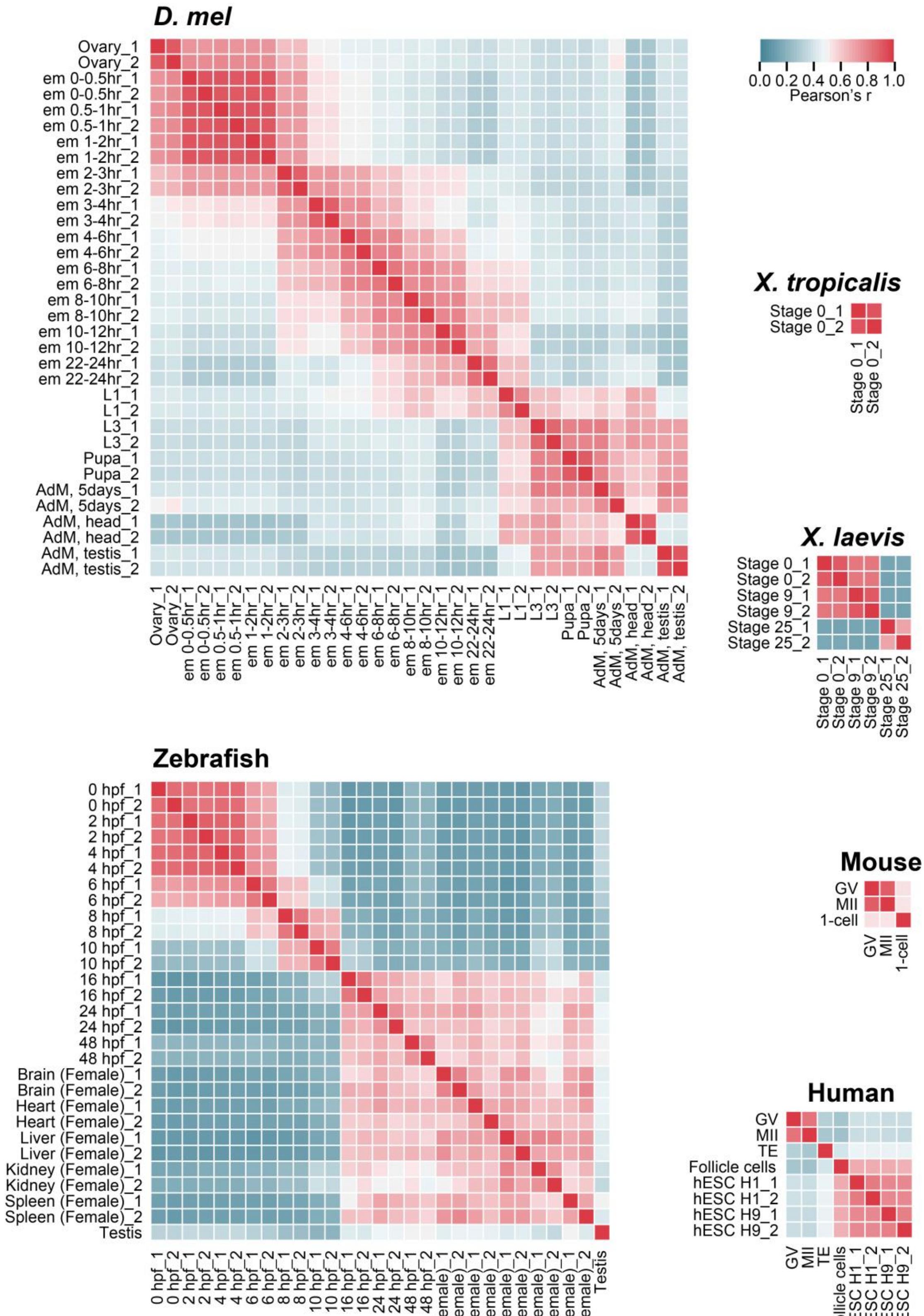

Supplementary Figure 1. Comparison of methylation levels between replicates.

Heatmap of Pearson correlations on the methylation levels of different samples in

different species. “_1” and “_2” mean biological replicate 1 and replicate 2. Source data are provided as a Source Data file.

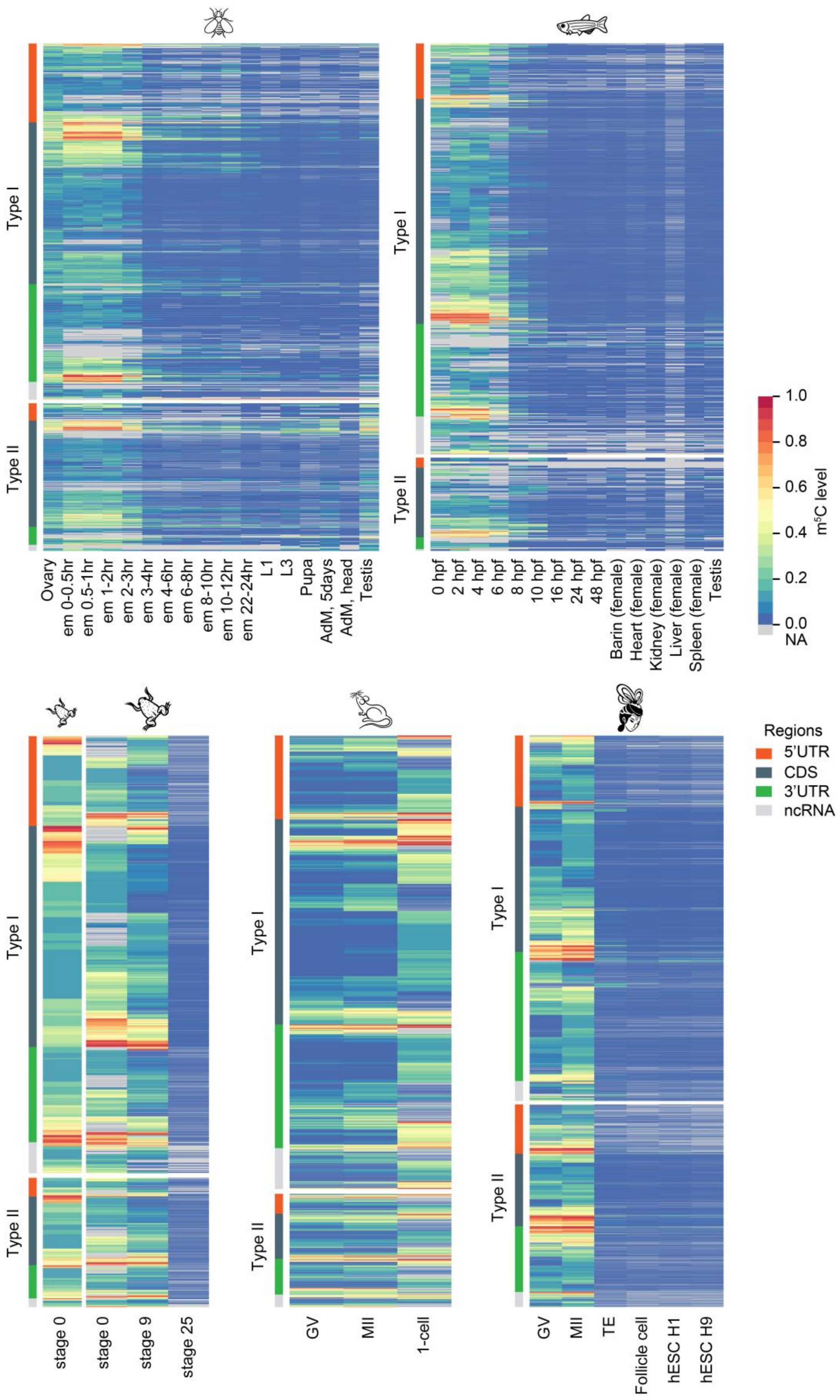

Supplementary Figure 2. Heatmap of m^{5}C levels in different species.

For each species, sites with methylation levels ≥ 10% in at least one sample were shown. For *X. tropicalis*, zebrafish, *D. mel*, and human ES cell samples, the m^5C level of a site was determined by combining replicates of a sample. Type I and Type II m^5C sites in different genic locations were shown separately. Notably, the heatmap height of Type I and Type II sites was adjusted for better visualization and thus was not proportional to the number of m^5C sites. Source data are provided as a Source Data file.

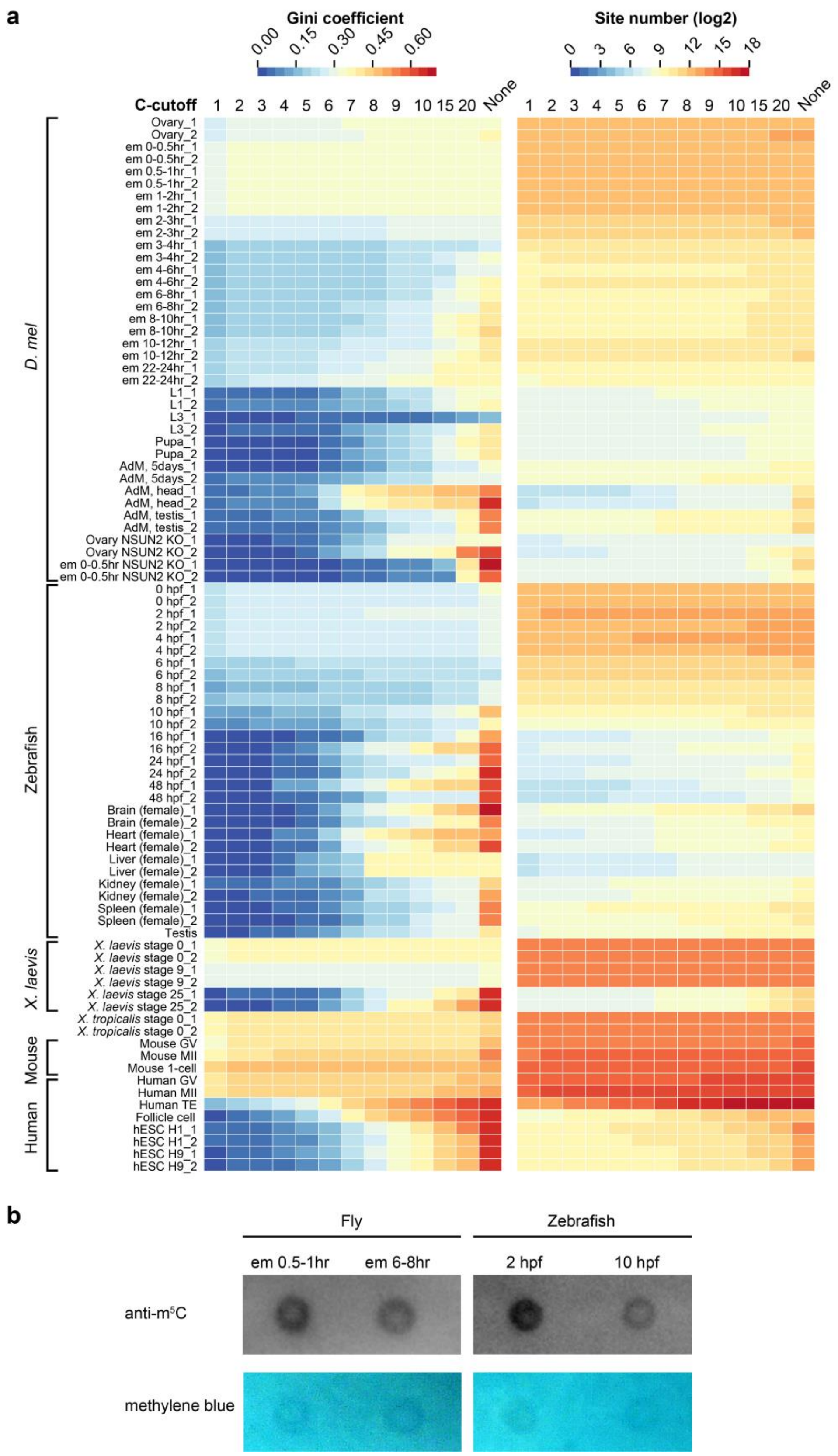

Supplementary Figure 3. The verification of the extensive methylation of maternal mRNAs and the low-level methylation of zygotic mRNAs.

a, The relationship between Gini coefficient, site number, and C-cutoff filter. **b**, Top: dot blotting on 75 ng polyadenylated RNAs with an anti-m^{5}C antibody (Diagenode, C15200003). Bottom: methylene blue staining of the RNAs served as a loading control. The results were consistent in two biological replicates. Source data are provided as a Source Data file.

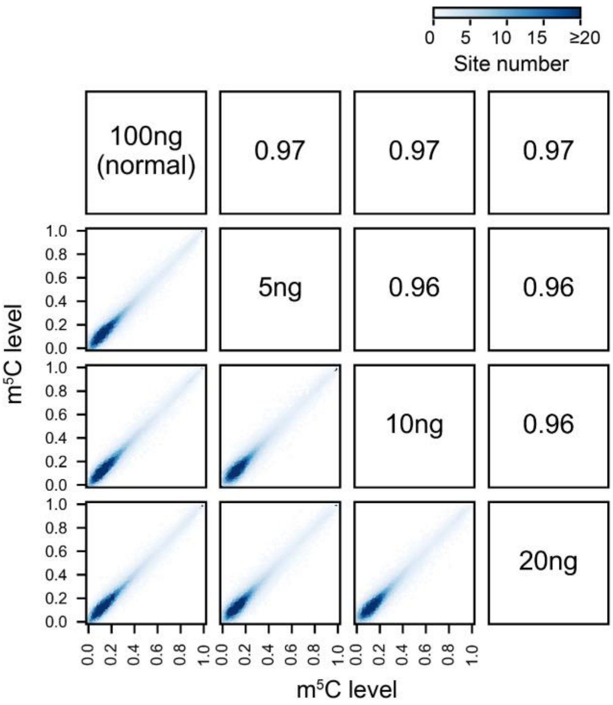

Supplementary Figure 4. Reproducibility of methylation level measurement using different amounts of RNA input.

Poly(A) selected RNAs isolated from *X. tropicalis* stage 0 embryos were used. In the pairwise comparison, sites that are covered by at least 50 reads in both samples and with methylation levels ≥ 10% in at least one sample were shown. Source data are provided as a Source Data file.

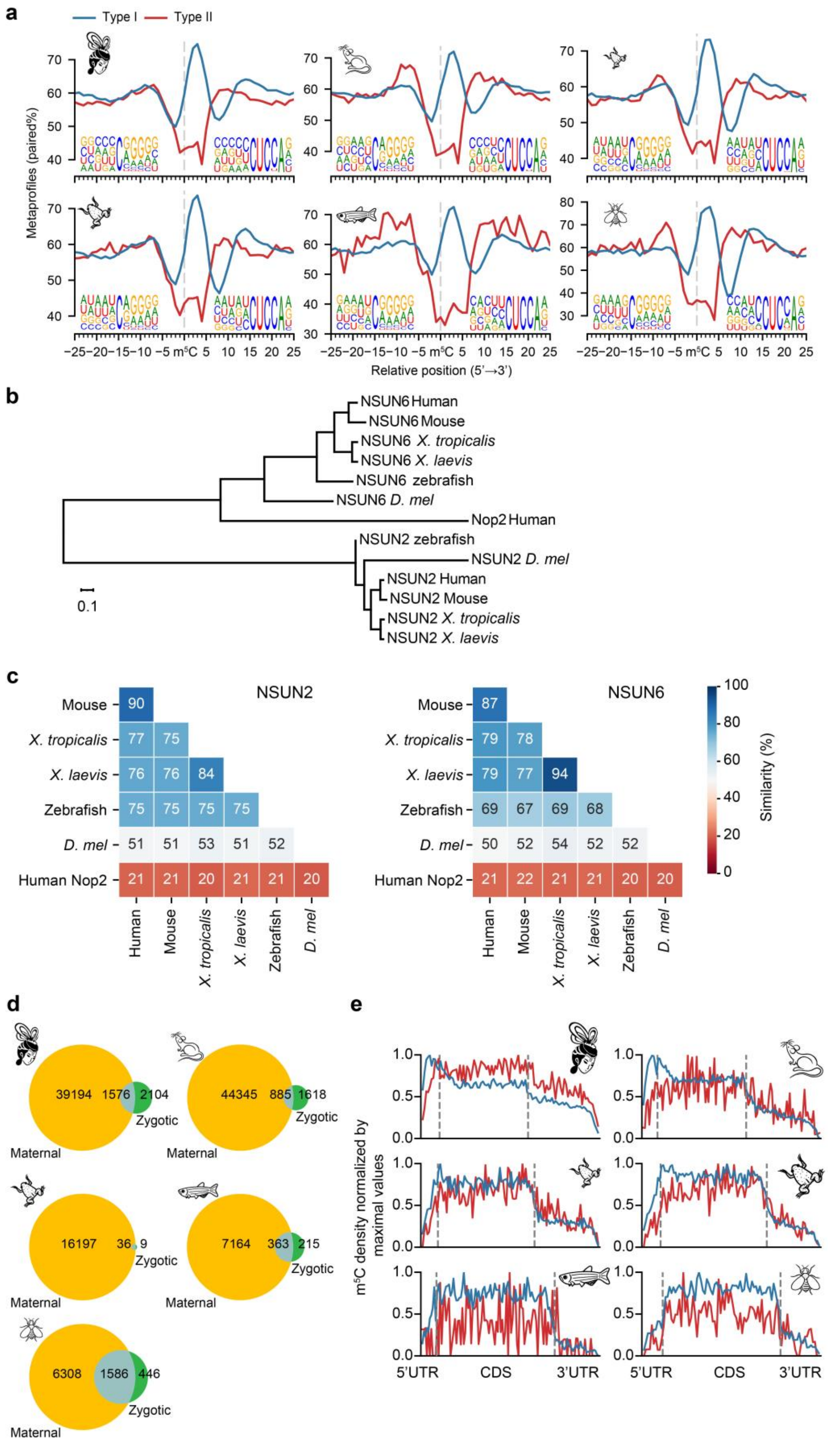

	Human	Mouse	X. tropicalis	X. laevis	Zebrafish	D. mel
**NSUN2**						
Mouse	90					
X. tropicalis	77	75				
X. laevis	76	76	84			
Zebrafish	75	75	75	75		
D. mel	51	51	53	51	52	
Human Nop2	21	21	20	21	21	20
**NSUN6**						
Mouse	87					
X. tropicalis	79	78				
X. laevis	79	77	94			
Zebrafish	69	67	69	68		
D. mel	50	52	54	52	52	
Human Nop2	21	22	21	21	20	20

Supplementary Figure 5. NSUN2- and NSUN6- mediated maternal mRNA m^{5}C methylation in different species.

a, The sequence motif and metaprofiles of the secondary structure of the maternal m^5C sites and flanking regions in different species. Maternal sites were defined as sites identified in stages with mRNAs largely transcribed by the maternal genome (underlined in **Fig. 1a**). The upstream and downstream 50 nt sequences of the m^5C sites were extracted from the transcriptome and folded with RNAfold, and the 25bp flanking regions were shown. Y-axis means the percentage of paired bases at each position. Motifs of Type I and Type II sites were shown on the left and right, respectively. **b,** Analysis was conducted using MEGA[1]. The amino acid sequences of NSUN2 and NSUN6 were obtained from Ensembl and aligned, and an evolutionary phylogenetic tree was inferred using the maximum likelihood method. **c**, Pairwise comparison showing the protein similarity of NSUN2 proteins between different species. **d**, Overlaps between maternal and zygotic mRNA m^5C sites in different species. Human zygotic sites, sites identified in H1, H9, TE in this study and in 7 adult tissues in our previous study[2]; mouse zygotic sites, sites identified in 9 adult tissues in our previous study[2]; zebrafish zygotic sites, sites identified in 10 hpf embryos, later developmental stages, and 6 adult tissues; *X. laevis* zygotic sites, sites identified in stage 25 embryos; *D. mel* zygotic sites, sites identified in 4-6 hour embryos, later developmental stages, and 3 adult samples. **e**, The normalized distribution of maternal m^5C sites along the transcripts in different species. In this analysis, to have a fair comparison of the Type I and Type II site distribution, the individual distributions in **Fig. 1d** were standardized with their maximum value as 1 separately. Source data are provided as a Source Data file.

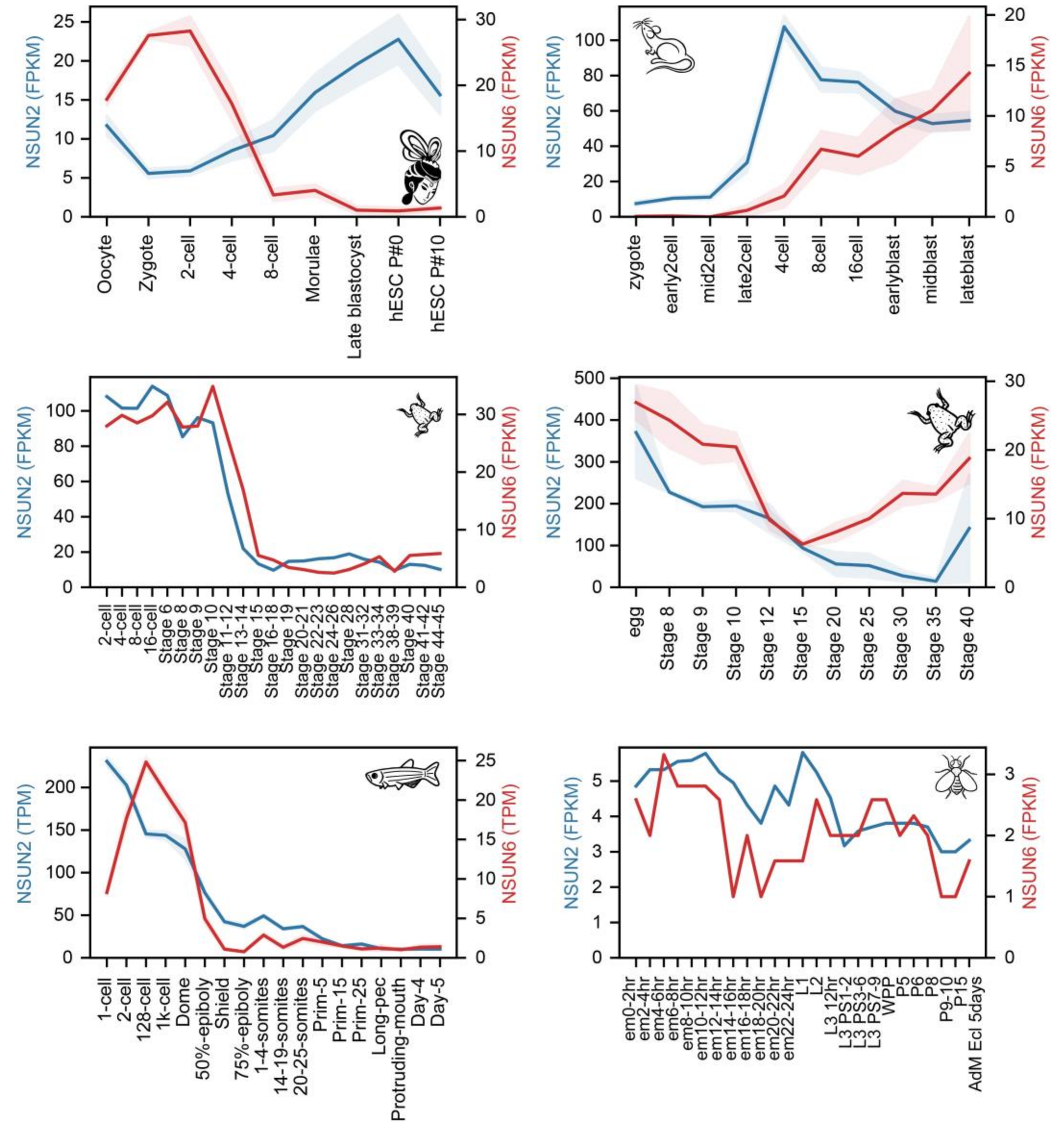

Supplementary Figure 6. NSUN2 and NSUN6 expression levels in different species.

Expression profiles of NSUN2 and NSUN6 mRNAs in species studied. Data were from developmental time course transcriptional profiling of *D. mel*, zebrafish, *X. laevis*, *X. tropicalis*, mice, and humans generated by modENCODE[3], White et al.[4], Session et al.[5], Tan et al.[6], Deng et al.[7], and Yan et al.[8], respectively. For studies with biological replicates in different developmental stages (zebrafish, *X. laevis*, mice, and humans), mean expression levels and SE were shown. Source data are provided as a Source Data file.

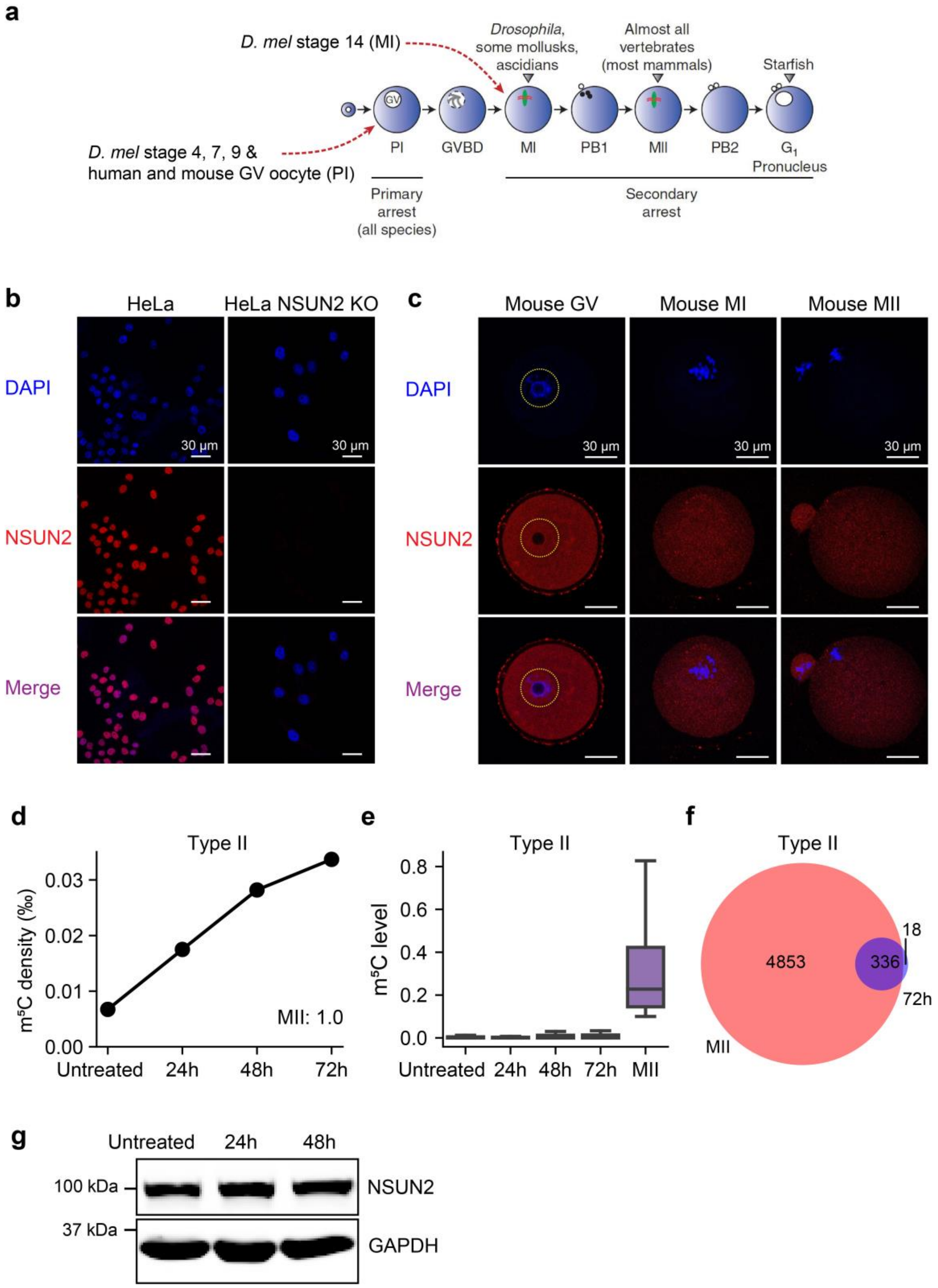

Supplementary Figure 7. The deposition of mRNA m^{5}C in oocytes.

a, Meiotic arrests during oocyte development in different species. Oocytes from most species undergo the first arrest at prophase I (PI) that is maintained for a few days (*Drosophila*) or for decades (humans). On hormonal or developmental stimulation,

oocytes undergo meiotic maturation, release the primary arrest, and enter the second arrest at metaphase I (MI), metaphase II (MII), or post meiotic G1 depending on the species. Stages we used for staining are indicated. GV, germinal vesicle; GVBD, germinal vesicle breakdown; PB1, polar body 1; PB2, polar body 2 (Adapted from Stetina et al., 2011[9]). **b**, Representative images of wild-type and NSUN2 knockout HeLa cells stained with anti-NSUN2 antibody. The localization of NSUN2 in wild-type cells was consistent under different fields of the microscope in two biological replicates. Scale bars, 30 μm. **c**, Representative images of mouse GV/MI/MII oocytes stained with anti-NSUN2 antibody. 15 GV, 27 MI, and 11 MII oocytes were stained, and the NSUN2 localization was consistent in all oocytes examined. Scale bars, 30 μm. **d-e**, The densities (**d**) and levels (**e**) of Type II m^5C sites in HeLa cells treated with nocodazole for 0, 24, 48, and 72 hours. Boxplots (n=1337): 25th to 75th percentiles (boxes), medians (horizonal lines), and 1.5 times of the interquartile range (whiskers). In panel **e**, a union of sites with levels ≥ 10% in at least one sample were used for analysis. The density of mRNA m^5C sites in MII oocytes is indicated in **d**. **f**, Overlaps of Type II mRNA m^5C sites between HeLa cells treated with nocodazole for 72 hours and MII oocytes. **g**, Western blotting showing the NSUN2 protein levels in untreated and treated HeLa samples. Source data are provided as a Source Data file.

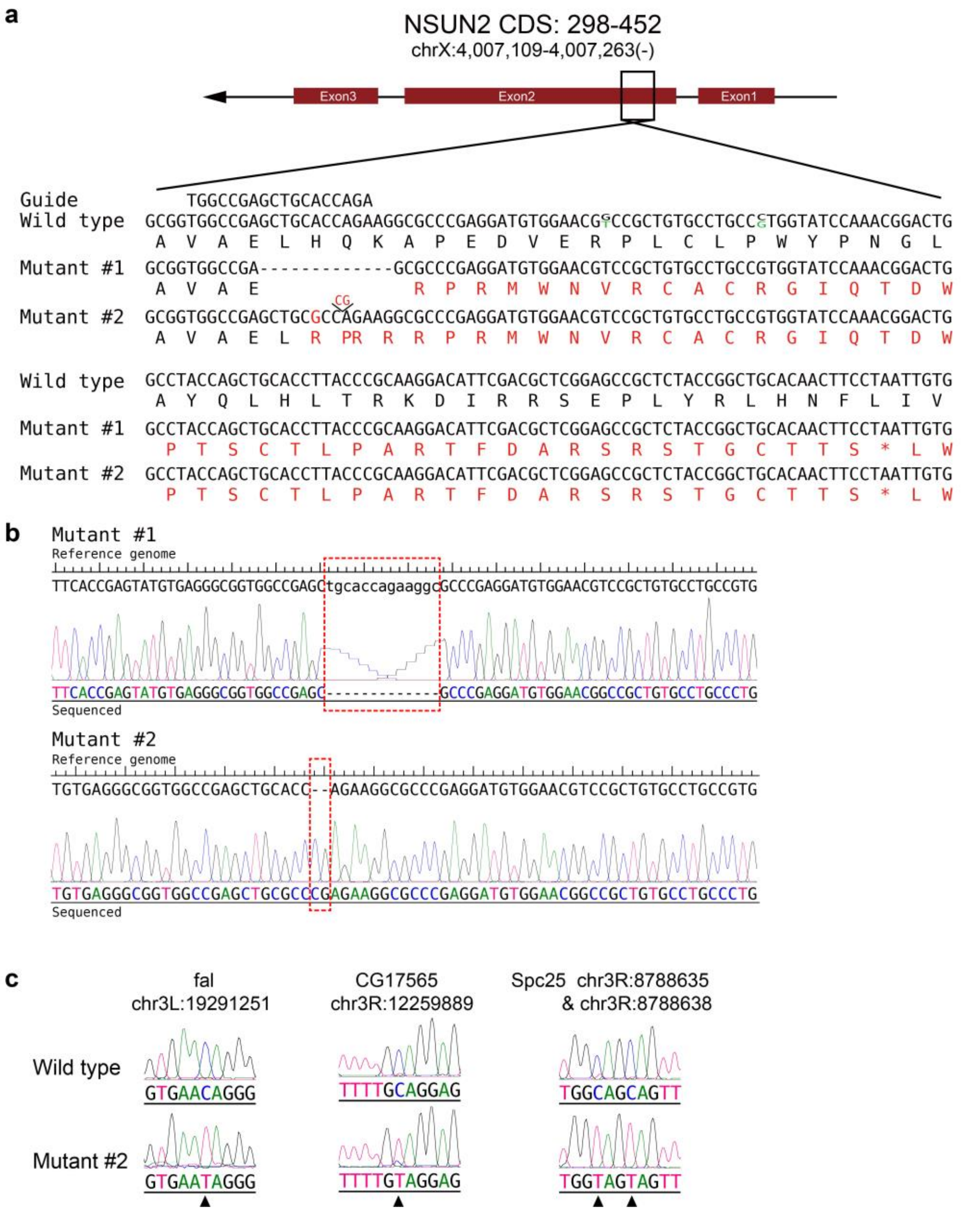

Supplementary Figure 8. The generation of NSUN2 knockout flies.

a, Schematic representation of NSUN2 mutant generation using the CRISPR/Cas9 system. The sequence of sgRNA and information for the mutants are indicated. Mutant #1 line has a frameshift deletion in NSUN2 CDS regions. Mutant #2 line has a two bp insertion. Both mutations result in an enzymatic dead truncated protein. Mutant #1 line was used for most of the experiments and analyses. **b**, Sanger trace showing the successful deletion in the mutant flies. **c**, Sanger trace of PCR products amplified using cDNAs derived from bisulfite-treated total RNAs. Total RNAs were from 0-0.5 hour wild-type and mutant #2 embryos. Four high-level m^{5}C sites were

selected and 3 primer pairs were designed based on C-to-T converted reference genes.

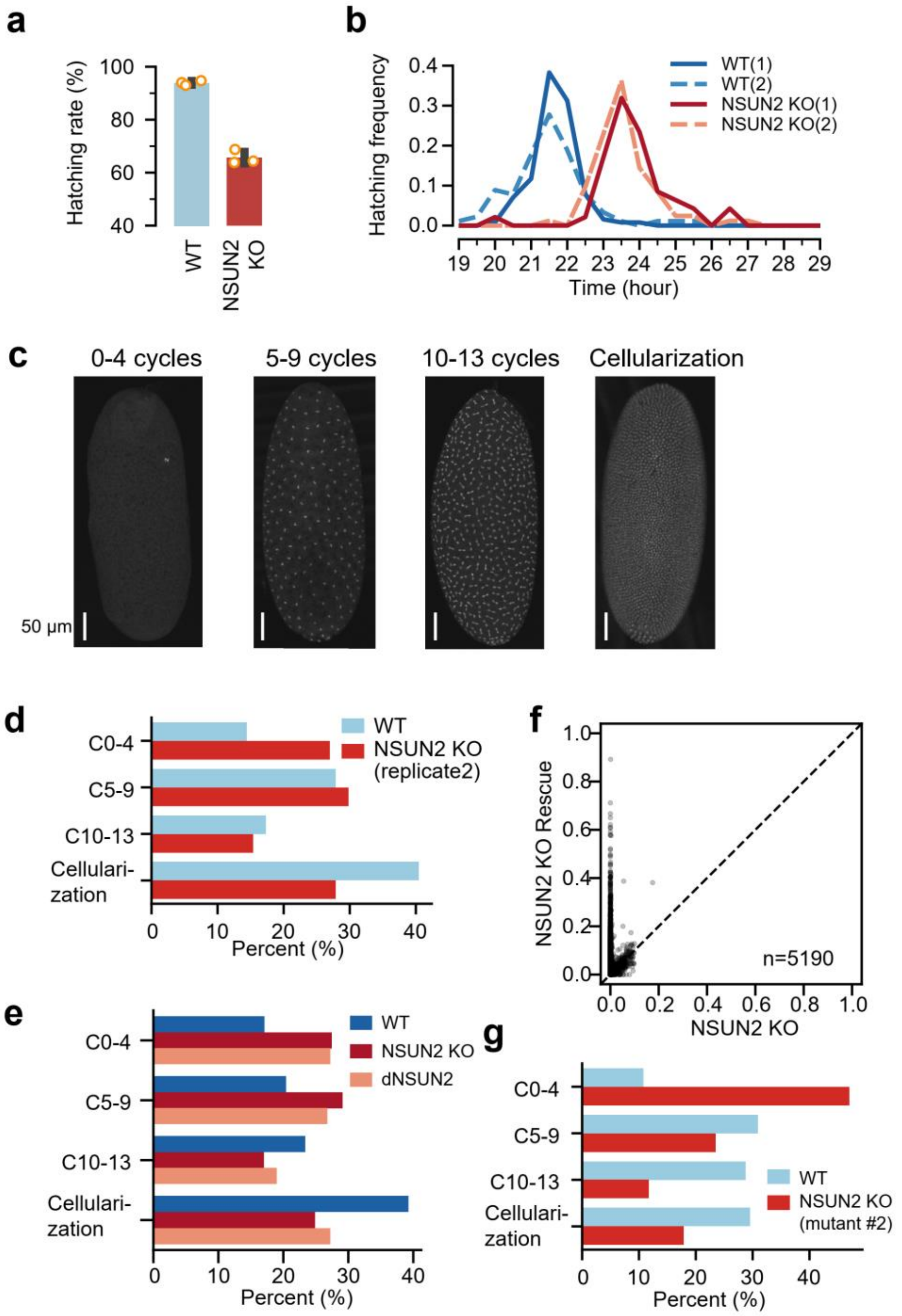

Supplementary Figure 9. The deficiency of NSUN2 in the embryo leads to a developmental delay.

a, The hatching rate of eggs laid by NSUN2 mutant females is reduced. Shown is the percentage of hatching embryos into larvae from female flies of indicated genotypes. Error bars represent standard deviation from three independent experiments. **b**, The times between oviposition and the hatching of first instar larvae. Two independent experiments were performed. **c**, Representative DAPI staining images within the first

2 hours of *D.mel* embryonic development. Wild-type embryos were used as examples: embryos that in 0-4 divisions, 5-9 divisions, 10-13 divisions, and cellularization. **d**, Quantification of the percentage of 0-2 hour embryos that were present in different cycles of embryogenesis. The second independent experiment of mutant #1 line was shown. **e**, Quantification of the percentage of 0-2 hour embryos that were present in different cycles of embryogenesis. dNSUN2 means NSUN2 mutant flies rescued by the catalytically inactive NSUN2 (C323A mutant, corresponding to the human C321A mutant[10]). **f**, Comparison of m^5C methylation levels in the ovaries between mutant #1 and rescued flies. **g**, Quantification of the percentage of 0-2 hour embryos that were present in different cycles of embryogenesis. The mutant #2 line was used for analysis. The numbers of flies used in each panel are provided in Source Data. Source data are provided as a Source Data file.

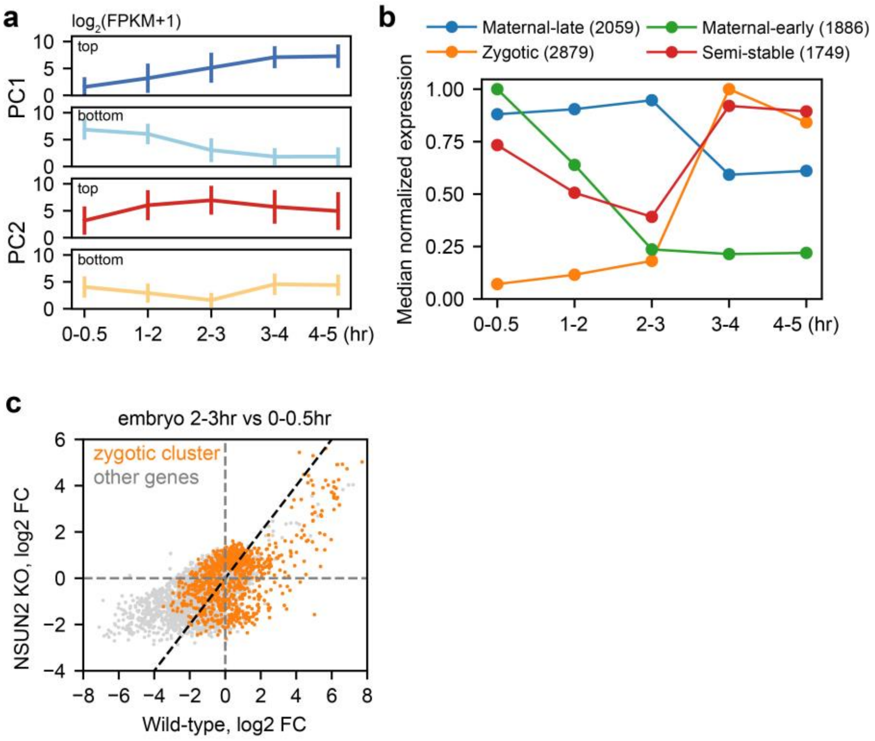

Supplementary Figure 10. Examining the gene expression profiles of wild-type and NSUN2 mutant embryos.

a, Average FPKM values and their standard deviations were shown for the top 100 and bottom 100 genes that contribute most to PC1 and PC2 across wild-type embryonic development (8573 genes were analyzed). **b**, Classification of *D. mel* genes during 0-5 hours of development. Genes with FPKM values >1 in at least one stage were used. FPKM of all genes were first normalized to the highest value of five points. Then k-Means clustering (k = 4) was conducted with Python module scikit-learn. **c**, Comparison of gene expression changes (2-3 hour embryos vs 0-0.5 hour embryos) of zygotic transcripts (2879 genes in **b**) between wild-type and maternal NSUN2 knockout embryos. Source data are provided as a Source Data file.

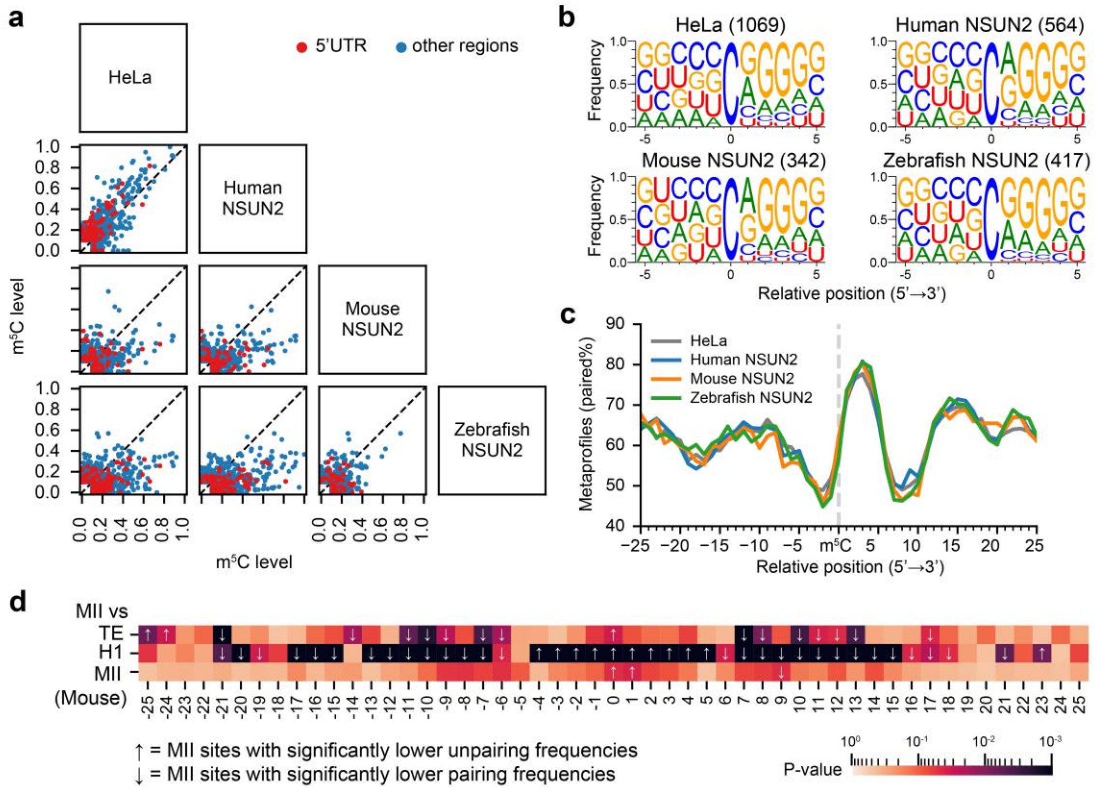

Supplementary Figure 11. Two major m^{5}C regulatory innovation steps from invertebrates to mammals and then humans.

a, Pairwise comparison of Type I sites identified in wild-type HeLa cells and NSUN2 knockout HeLa cells expressing human, mouse, and zebrafish NSUN2 individually. Only sites that are covered by at least 20 reads in both samples were analyzed. **b-c**, Type I sites methylated by mouse and zebrafish NSUN2 showed sequence (**b**) and structural preferences (**c**) similar to those methylated by human NSUN2. **d**, Comparison of the structural difference of Type II m^{5}C sites and flanking regions between human MII oocytes and other samples (hESC H1, human TE, and mouse MII oocytes). The bootstrapping method was used and p values were calculated using one-sided student's t-test (**Methods**). Positions in human MII oocytes with significantly lower unpairing frequencies (loop region) and lower pairing frequencies (stem region) than other samples are indicated with up and down arrows, respectively. Source data are provided as a Source Data file.

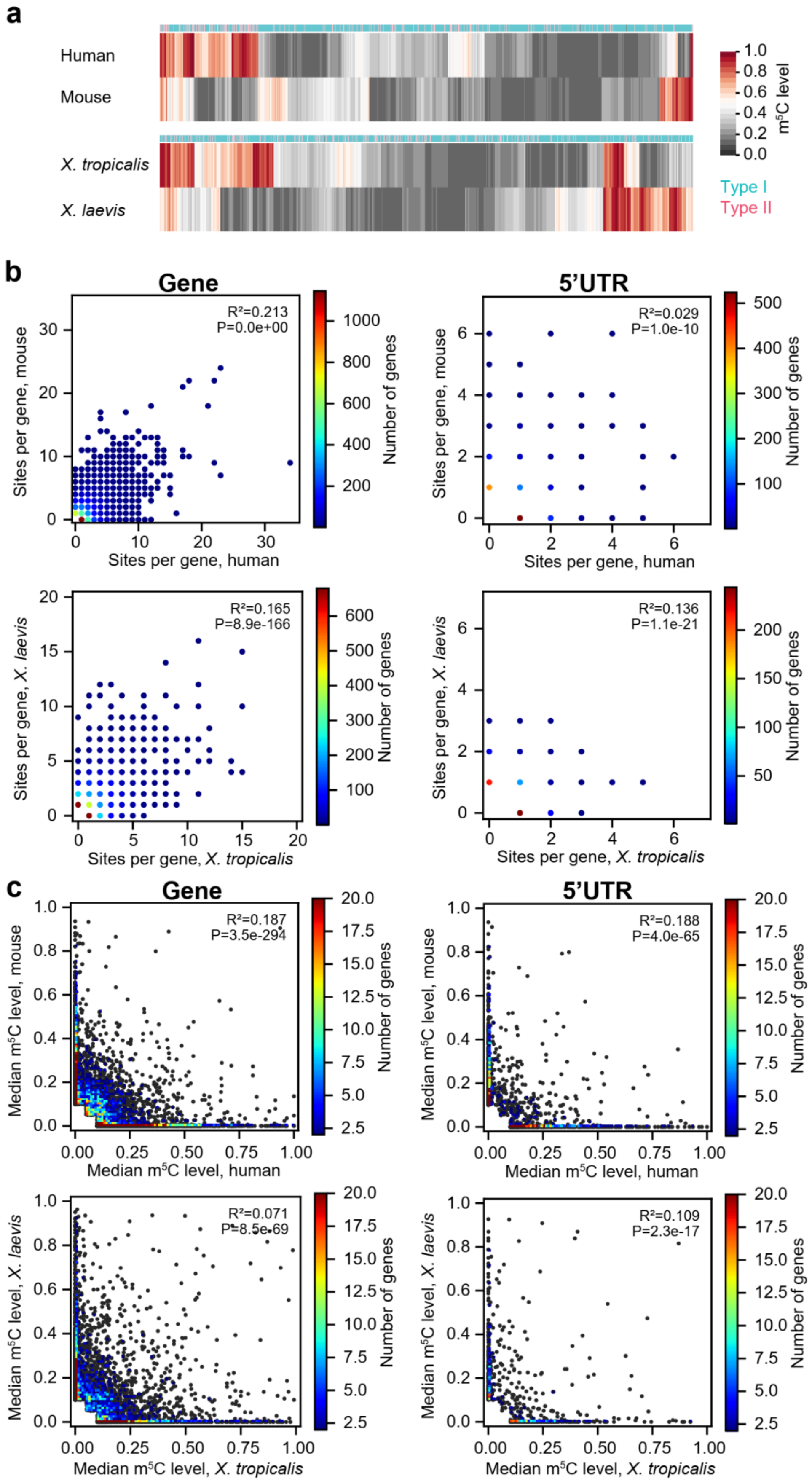

Supplementary Figure 12. mRNA m^{5}C evolution in human-mouse pair and *X.*

***laevis*-*X. tropicalis* pair.**

a, Comparison of methylation levels in human-mouse pair and *X. laevis*-*X. tropicalis* pair. For humans and mice, methylation levels measured from MII oocytes were used. For frog species, methylation levels measured from stage 0 embryos were used. Sites that are covered by ≥ 20 reads in both species were analyzed. **b-c**, Scatterplot showing the m^5C site number (**b**) or median m^5C levels (**c**) of orthologous genes or 5'UTR of orthologous genes in human-mouse pair and *X. laevis* -*X. tropicalis* pair. For humans and mice, methylation sites or levels obtained from MII oocytes were used. For frog species, methylation sites or levels obtained from stage 0 embryos were used. Sites that are covered by ≥ 20 reads in both species were analyzed. Source data are provided as a Source Data file. The Person's R^2 and the P-values are indicated.

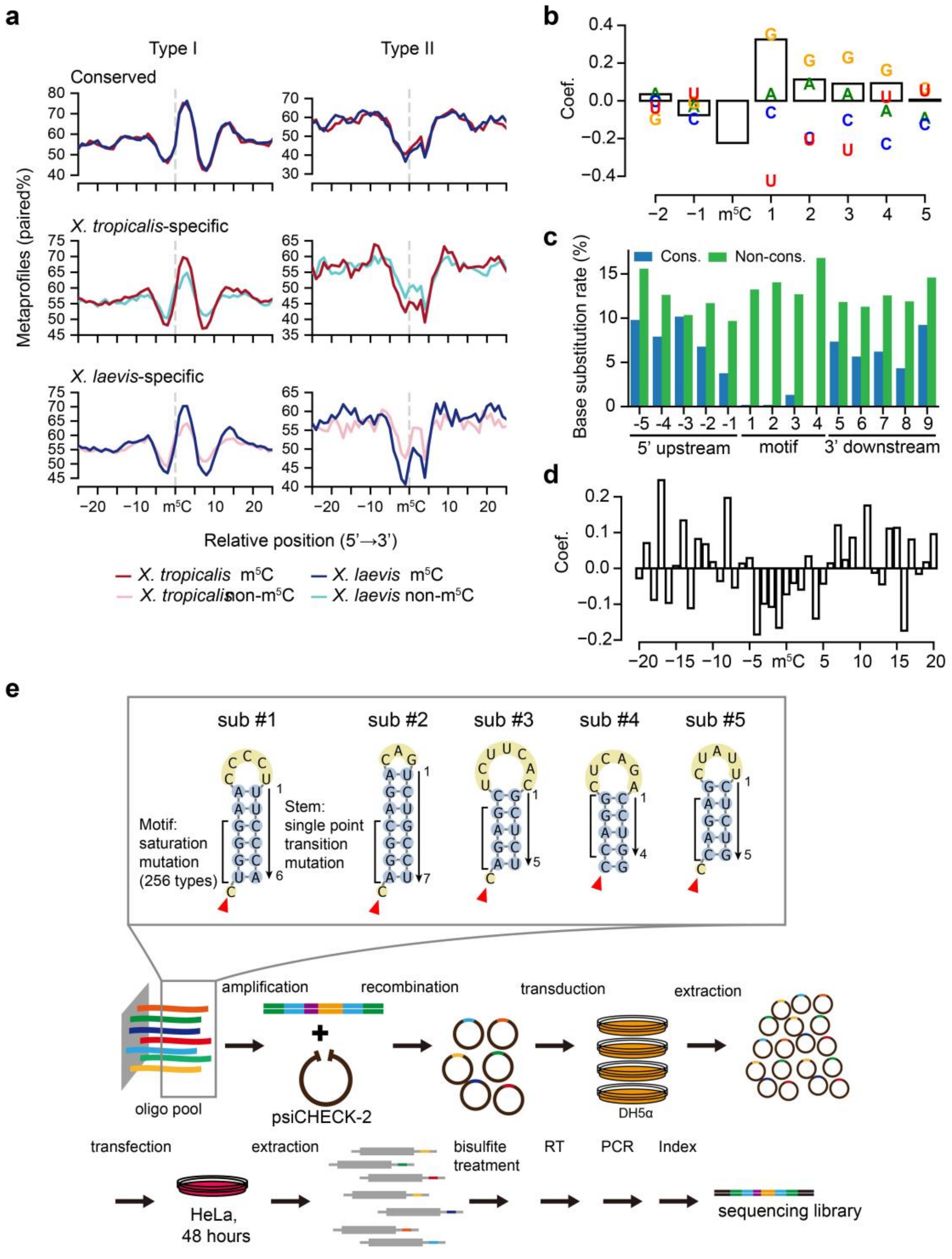

Supplementary Figure 13. Sequence- and structure- dependent evolution of mRNA m^{5}C.

a, The metaprofiles of the secondary structure for conserved and non-conserved Type I and Type II m^{5}C sites between *X. tropicalis* and *X. laevis*. Conserved, m^{5}C sites that were methylated in both species. *X. tropicalis*-specific and *X. laevis*-specific, m^{5}C

sites that were Cs at the DNA level in both species but only methylated in *X. tropicalis* or *X. laevis*. Samples used: stage 0 embryos. **b**, GLM coefficients of sequence and structural factors for the gain or loss of Type I sites between *X. tropicalis* and *X. laevis* (see **Methods**). Analysis was performed as in **Fig. 5c**. **c**, Base substitution rates in the motif and flanking regions of conserved and non-conserved Type II sites between *X. tropicalis* and *X. laevis*. **d**, GLM coefficients of structural feature for the gain or loss of Type II sites between *X. tropicalis* and *X. laevis*. **e**, Top: The predicted secondary structure of substrates. The m^5C site is indicated by the red arrowhead. 5 Type I m^5C sites with moderate or high methylation levels in HeLa cells were selected. On the basis of these targets, we designed sequence variants to systematically impact the sequence motif and secondary structure surrounding the m^5C sites. Bottom: The schema of the methylation reporter assay. Each of these sequences further comprised a barcode and common adapters on both ends. The sequences were synthesized as DNA oligo pools, cloned as 3’UTR elements downstream of a luciferase gene, and transfected into wild-type HeLa cells. Their methylation levels were determined by targeted BS-seq. Source data are provided as a Source Data file.

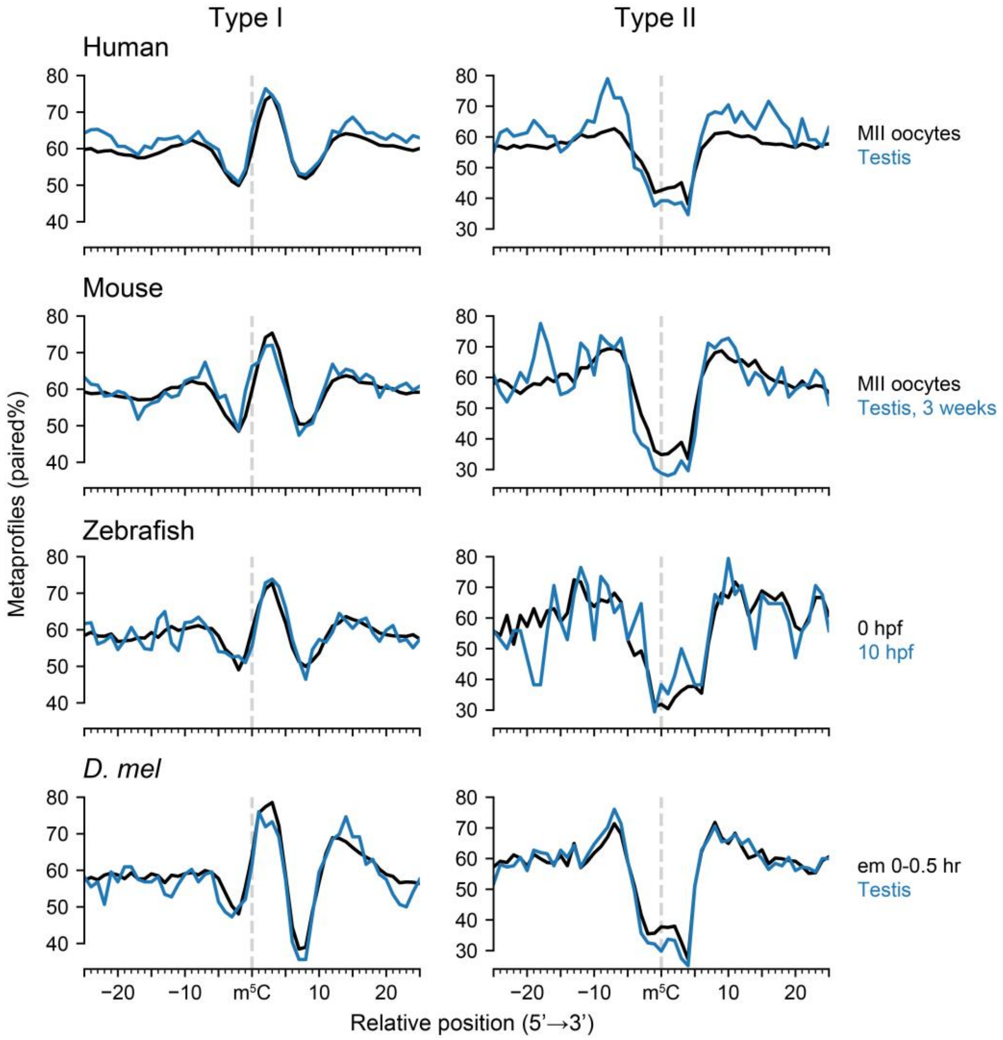

Supplementary Figure 14. The metaprofiles of the secondary structure for maternal and zygotic Type I and Type II m^{5}C sites.

For each species, two samples that represent maternal and zygotic mRNA m^{5}C patterns were selected. *X. laevis* was excluded because only 45 zygotic sites were identified. Since NSUN6 had a relatively high expression in human, mouse, and *D. mel* testis[11], to obtain enough zygotic Type II sites for analysis, testis samples in these species were selected. Source data are provided as a Source Data file.

Supplementary Discussion. The impact of the X chromosome location of NSUN2 on phenotype assessment of the mutant flies.

NSUN2 is located in the X chromosome of the *D.mel* genome. When we crossed NSUN2 mutant virgins with wild-type males to produce maternal NSUN2 knockout embryos, all male embryos were homozygous mutants and all female embryos were heterozygous mutants. For Supplementary Fig. 9a, we cannot exclude the possibility that the observed lethality may be due to the zygotic loss of NSUN2 protein in male embryos. For Supplementary Fig. 9b, since nearly all mutant embryos were developmentally delayed, it is clear that the maternal loss of NSUN2 protein in female embryos led to a developmental delay. For Fig. 2g, Supplementary Fig. 9d and Supplementary Fig. 9g, since the C0-9 division stages barely have zygotic proteins expressed[12], including zygotic NSUN2, it is clear that the maternal loss of NSUN2 protein led to a developmental delay in both male and female embryos.

Supplementary References

1. Tamura, K., Stecher, G. & Kumar, S. MEGA11: Molecular Evolutionary Genetics Analysis version 11. *Mol Biol Evol* (2021).
2. Huang, T., Chen, W., Liu, J., Gu, N. & Zhang, R. Genome-wide identification of mRNA 5-methylcytosine in mammals. *Nat Struct Mol Biol* **26**, 380-388 (2019).
3. Graveley, B.R. *et al.* The developmental transcriptome of Drosophila melanogaster. *Nature* **471**, 473-479 (2011).
4. White, R.J. *et al.* A high-resolution mRNA expression time course of embryonic development in zebrafish. *eLife* **6** (2017).
5. Session, A.M. *et al.* Genome evolution in the allotetraploid frog Xenopus laevis. *Nature* **538**, 336-343 (2016).
6. Tan, M.H. *et al.* RNA sequencing reveals a diverse and dynamic repertoire of the Xenopus tropicalis transcriptome over development. *Genome research* **23**, 201-216 (2013).
7. Deng, Q., Ramskold, D., Reinius, B. & Sandberg, R. Single-cell RNA-seq reveals dynamic, random monoallelic gene expression in mammalian cells. *Science* **343**, 193-196 (2014).
8. Yan, L. *et al.* Single-cell RNA-Seq profiling of human preimplantation embryos and embryonic stem cells. *Nat Struct Mol Biol* **20**, 1131-1139 (2013).
9. Von Stetina, J.R. & Orr-Weaver, T.L. Developmental control of oocyte maturation and egg activation in metazoan models. *Cold Spring Harb*

Perspect Biol **3**, a005553 (2011).
10. King, M.Y. & Redman, K.L. RNA methyltransferases utilize two cysteine residues in the formation of 5-methylcytosine. *Biochemistry* **41**, 11218-11225 (2002).
11. Liu, J. *et al.* Sequence- and structure-selective mRNA m5C methylation by NSUN6 in animals. *National Science Review*, Published online October 31, 2020. (2020).
12. Lee, M.T., Bonneau, A.R. & Giraldez, A.J. Zygotic Genome Activation During the Maternal-to-Zygotic Transition. *Annual review of cell and developmental biology* **30**, 581-613 (2014).
