# Supplementary material for: Developmental mRNA m5C landscape and regulatory innovations of massive m5C modification of maternal mRNAs in animals
Source: Nat Commun. 2022 May 5;13:2484. doi: 10.1038/s41467-022-30210-0 (PMC9072368; doi:10.1038/s41467-022-30210-0)
Supplement: Supplementary file 3 — Description of Additional Supplementary Files [file 41467_2022_30210_MOESM3_ESM.pdf]

**Description of Additional Supplementary Files**

File Name: Supplementary Data 1
Description: RNA BS-seq and RNA-seq library summary.

File Name: Supplementary Data 2
Description: m5C site list.

File Name: Supplementary Data 3
Description: Motifs identified via MEME.

File Name: Supplementary Data 4
Description: Expression profile data in D. mel.

File Name: Supplementary Data 5
Description: NSUN2 substrate mutagenesis analysis.
