# Supplementary figures and images for: Developmental mRNA m5C landscape and regulatory innovations of massive m5C modification of maternal mRNAs in animals
Source: Nat Commun. 2022 May 5;13:2484. doi: 10.1038/s41467-022-30210-0 (PMC9072368; doi:10.1038/s41467-022-30210-0)

## Slide 1
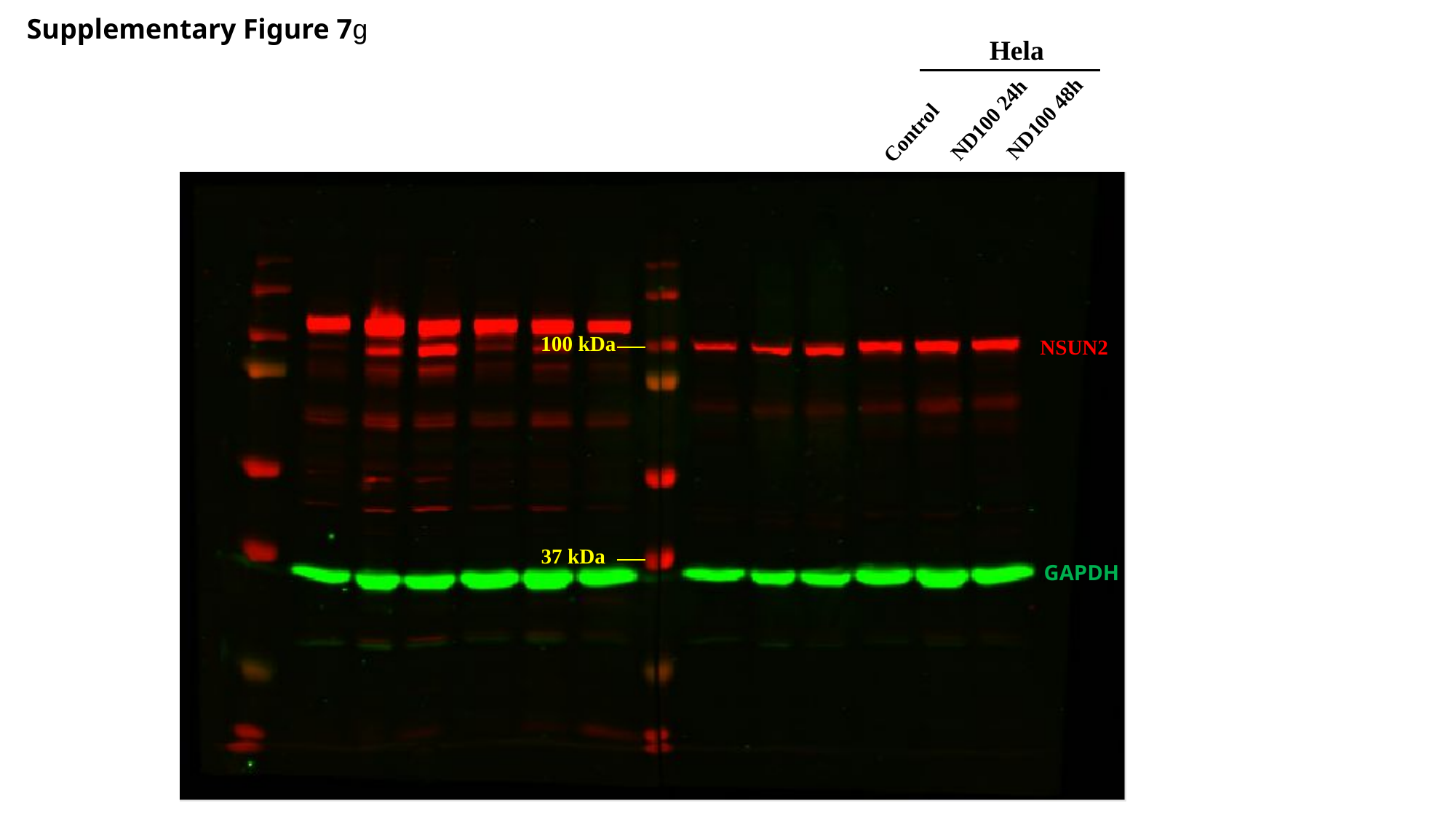

Supplementary Figure 7g
Hela
ND100 48h
ND100 24h
Control
100 kDa
NSUN2
37 kDa
GAPDH

Supplement: Supplementary file 10 — Source Data [file 41467_2022_30210_MOESM10_ESM.zip › Source Data/Supplementary Figure 7g-unprocessed scans.pptx]
